# Assessment of River Habitat Quality in the Hai River Basin, Northern China

**DOI:** 10.3390/ijerph120911699

**Published:** 2015-09-17

**Authors:** Yuekui Ding, Baoqing Shan, Yu Zhao

**Affiliations:** 1 State Key Laboratory on Environmental Aquatic Chemistry, Research Center for Eco-Environmental Science, Chinese Academy of Science, Beijing 100085, China; E-Mails: yuekuiding@163.com (Y.D.); zhaoyugreen@163.com (Y.Z.); 2 University of Chinese Academy of Science, Beijing 100049, China

**Keywords:** assessment, river habitat quality, Hai River Basin, indicator, scoring system, impact factor

## Abstract

We applied a river habitat quality (RHQ) assessment method to the Hai River Basin (HRB); an important economic centre in China; to obtain baseline information for water quality improvement; river rehabilitation; and watershed management. The results of the assessment showed that the river habitat in the HRB is seriously degraded. Specifically; 42.41% of the sites; accounting for a river length of 3.31 × 10^4^ km; were designated poor and bad. Habitat in the plain areas is seriously deteriorated; and nearly 50% of the sites; accounting for a river length of 1.65 × 10^4^ km; had either poor or bad habitats. River habitat degradation was attributable to the limited width of the riparian zone (≤5 m); lower coverage of riparian vegetation (≤40%); artificial land use patterns (public and industrial land); frequent occurrence of farming on the river banks and high volumes of solid waste (nearly 10 m^3^); single flow channels; and rare aquatic plants (≤1 category). At the regional scale; intensive artificial land use types caused by urbanization had a significant impact on the RHQ in the HRB. RHQ was significantly and negatively correlated with farmland (r = 1.000; *p* < 0.01) and urban land (r = 0.998; *p* < 0.05); and was significantly and positively correlated with grassland and woodland (r = 1.000; *p* < 0.01). Intensive artificial land use; created through urbanization processes; has led to a loss of the riparian zone and its native vegetation; and has disrupted the lateral connectivity of the rivers. The degradation of the already essentially black rivers is exacerbated by poor longitudinal connectivity (index of connectivity is 2.08–16.56); caused by reservoirs and sluices. For river habitat rehabilitation to be successful; land use patterns need to be changed and reservoirs and sluices will have to be regulated.

## 1. Introduction

River habitat has been defined as the local physical, chemical and biological features that provide environments for instream biota [1]. It is important to assess the physical river habitat when evaluating river health [2]. In addition, recreating and restoring the river habitat for ecosystems is a key step in river restoration [3]. In recent years, due to increasing awareness of the importance of river health and the increased application of habitat evaluations, many studies documenting a range of habitat assessment methods have been published. A brief review of this literature shows that methods for evaluating river habitat can generally be classified into two groups. The first group involves partial to macro-scale evaluation and mapping, often by means of remote sensing techniques [4,5,6]. This approach is based on geographic information system (GIS) spatial analysis and remote sensing image data. To select and to extract key habitat environmental factors affecting organisms, and habitat quality is determined through spatial analysis at regional scale. The second approach relies on field surveys and is based on field data collection. Indicators surveyed often include channel attributes, riparian characteristics, and land-use adjacent to river which is easily observed and measured at a site. Using a scoring system, different components are integrated in these RHQ assessments. For instance, the River Habitat Survey (RHS) used in the EU [7], the Riparian, Channel, and Environmental Inventory (RCE) used in Sweden [8], the Rapid Bioassessment Protocol used in the USA [9], and the Index of Stream Condition (ISC) used in Australia are based on different components and use different scoring systems [10]. Comparison of the two approaches shows that the second method is more straightforward and provides more detail about RHQ at the reach scale; it may not, however, consider all aspects of river condition, but rather has a focus on certain characteristics. For instance, Munné *et al.* [11] developed a new habitat quality indicator for rivers and streams (QBR index) based on four components of riparian habitat: total riparian vegetation cover, cover structure, cover quality and channel alterations. In conclusion, scoring systems, based on field surveys at the reach scale, are useful tools for river restoration and management, and are more useful than methods based on mapping.

The Hai River Basin (HRB), located in northern China, is an important political, economic, and cultural centre of China, and it includes the highly-developed cities of Beijing and Tianjin, both of which have an important role in the continued development of the national economy. The rate of urbanization in the HRB has reportedly increased rapidly in recent decades, from 18% in 1978 to 46% in 2009 [12,13], which has direct implications for water quality [14]. The river system has been important in supporting the lives and livelihoods of the local populace through its role in food provision, providing water for industry and agriculture, recreation, shipping and commerce [15]. However, with the acceleration of economic development, river pollution has increased, to the extent that the HRB is now one of the most polluted basins in China. Many of its rivers, especially in the plain area, experience acute water shortages and water pollution [16,17]. In addition, human activities, such as cultivation, irrigation, deforestation, and water conservancy projects have seriously disrupted the hydrological cycle and the spatial and temporal distribution of water resources [18]. In short, the combination of the rapid urbanization and population growth, industrial development, and the disruption of the natural hydrologicalcycle, has contributed to serious degradation of the river habitat in the HRB.

Fortunately, the government made a huge effort, and provided resources, to fight water pollution in the 12th Five Year Plan (2011–2015). Given its national importance and its severely degraded status, some of this pollution reduction activity, including restoration, was centred in the HRB. River rehabilitation and restoration could not be started until information about the RHQ of the HRB was available, but up until that point, no relevant studies or projects had been carried out. To what extent and in what aspects does river habitat degrades? How about spacial distribution of different river habitat quality? In response to a government request therefore, a scoring system, which is more suitable for river restoration and management (stated as above), was used to assess river habitat condition and to determine aspects of habitat degradation. Suitable indicators were selected to include in field surveys with which to evaluate the RHQ of the HRB and to determine the factors that control the RHQ. This research would lay a solid foundation for river restoration and management in the HRB.

## 2. Methods

### 2.1. Study Area and Sampling Sites

The Hai River Basin (HRB) is located between 112°–120° E and 35°–43° N, and covers an area of 3.18 × 10^5^ km^2^. It is bounded by the Mongolian Plateau to the north, the Yellow River to the south, the Taihang Mountains to the west, and Bohai Bay to the east. Landforms in the HRB include mountains, plateau, basins, and plains. Generally, there are large areas of mountains and plateau in the northwest, while broad plains dominate in the southeast. The HRB spans two climate types: semi-arid temperate and temperate monsoon climates. The annual average temperature in the basin ranges from 0 to 14 °C. The annual average precipitation is 547 mm, 75%–85% of which occurs from June to August.

**Figure 1:**
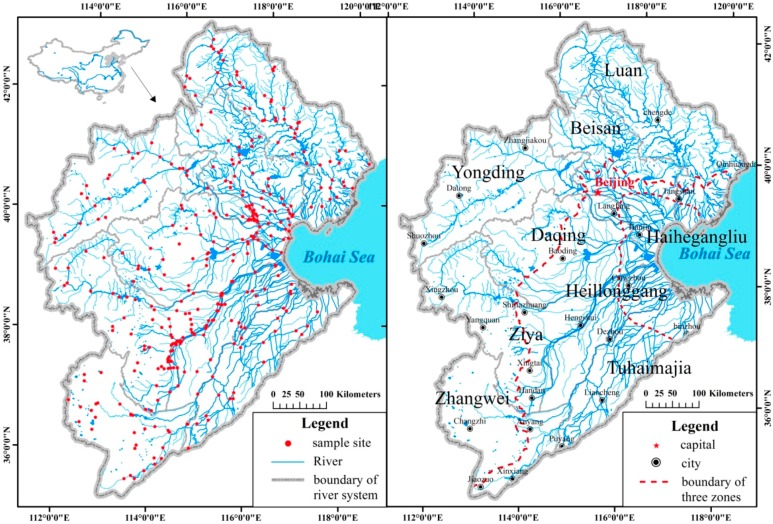
River system and sampling sites in Hai River Basin.

The HRB includes nine secondary river systems, the Luan, Beisan, Yongding, Daqing, Ziya, Heilonggang, Zhangwei, Tuhaimajia, and Haiheganliu (Figure 1). The Luanhe River, located in the north of the HRB, consists of two rivers, the Luanhe and Jidong rivers. Most of this catchment is in the mountains (85%). The remaining area is on the Inner Mongolia Plateau and the coastal plain. The Beisan River basin covers the Jingjinji Zone (Beijing–Tianjing–Hebei), China’s important economic growth pole. The Daqing River basin is in the middle of the HRB. Nearly half (48%) of its terrain is mountain land, and there are also relatively large areas of wetland, such as Baiyang Lake, in the plain area. The Yongding River basin is in the west of the HRB, next to the Daqing River basin. Most (93%) of the basin is in the mountains, and a small part is on the Inner Mongolian Plateau. The Ziya River basin is in the south of the HRB, of which 64% can be categorized as mountain area. The lowlands of the Ziya River are among the most polluted areas in the HRB. The Heilonggang River basin is to the east of the Ziya River basin, and is located in plain and littoral areas and has no mountains. Analogously, the Tuhaimajia River basin is also in the plain and littoral areas, and has a simple river system. The Zhangwei River basin, 63% of which is mountainous, is in the southwest corner of the HRB. The Haiheganliu in Tianjin City is the main flood discharge channel for the above river systems.

The rivers in the HRB can be classified into three physiographic sections, including the upper mountain area, the middle plain area, and the littoral area (Figure 1). The boundaries of the sections were marked according to “The Editorial Board of the Hai River (1997)”. The average river gradients in the upper mountain, middle plain, and littoral physiographic sections are 1‰–20‰, 0.1‰–10‰, 0‰–1‰ respectively. The littoral area is distinct from the plain area; the rivers in the littoral area are influenced by tidal interactions and have unique characteristics that make them different from the rivers in the middle plain area.

Each secondary river system is self-contained within the HRB. Sampling sites were selected within each secondary river system based on the following principles: (1) Sampling sites covered 2nd–4th order rivers. (2) Sampling sites included, where possible, existing hydrological monitoring sites to continue long-term monitoring. (3) To take account of the area of the river systems and the complexity of the river networks, river systems with larger areas had more sampling sites, and river systems with smaller areas and simple river networks had fewer sites. (4) Sampling sites were set up on both major and branch channels to study the differences and relationships between them. (5) Sites were located upstream and downstream of sewage outlets. (6) Comparatively fewer sites were set up on reaches with uniform characteristics. A total of 410 sampling sites were set up in the HRB, which covered a river length of 3.31 × 10^4^ km. The field survey was completed over a period of 3 months (from May to August, 2013) (Figure 1).

### 2.2. Indicators and Field Survey

#### 2.2.1. Indicators Selection

In general, the river habitat investigation covered features of the bank, in-stream, and adjacent land use [7,19,20]. Ten indicators were selected from the characteristics that were impacted most by human activity, including the width of the riparian zone, land-use patterns beyond the immediate riparian zone, riparian vegetation coverage, continuity of riparian vegetation, bank modification, channel structure, channel substrate, channel pattern, hydrophyte richness, and coverage of emergent plants along the channel (Table A1).

#### 2.2.2. Scoring and Assessment

While scoring systems are widely used to evaluate the relative quality of a site, they do have some disadvantages [7]. Following the River Habitat Survey (RHS) and the Riparian, Channel, and Environmental Inventory (RCE) [7,8], a scoring system was used to evaluate the RHQ in the HRB. In the assessment process basic principles were followed and scores were allocated as follows.

##### Low Pressure

Pressure on river habitats mainly derives from human activities in the HRB. As such, sites that experienced lower human pressure were allocated a high score. This was particularly reflected in the land use pattern and the width of the riparian zone adjacent to the river. Natural and near-natural land use, such as mountain vegetation and natural grassland, had higher scores, while artificial land use, for example, farmland, public land, and industrial land, had lower scores. The sequence of land use patterns followed the CORINE Land Cover (CLC) classification [21]. To provide a suitable habitat area, the riparian zone should be between 30 m and 500 m wide [22]. Because of human occupation, the maximum width of the riparian zone width in the HRB was 30 m.

##### Habitat Diversity

High habitat diversity, a positive feature that follows effective river restoration [23], contributes to increased biodiversity. The indicators of habitat diversity included channel substrate, channel pattern, and hydrophyte richness. Features that favoured habitat diversity also received a high score.

##### Continuity of Vegetation

Bank vegetation is an important component of physical habitat that can provide shade for aquatic life both through its effects on flow and its capacity. It also has a significant influence on bank stability [24,25,26]. High continuity expressed as a good coverage of bank vegetation received a high score. The rule was similarly applied for aquatic plants. However, particular note was made of river reaches where extensive instream macrophytes were obviously hindering flow. These reaches were assigned only one point because the excessive macrophyte growth was probably caused by eutrophication and pollution.

##### Stability and Cleanliness of the Channel

Waste dumped on the bank and in the channel increases the risk of water pollution and creates a stain on landscape aesthetics. Large quantities of solid waste that are left on the channel bank for a long time can significantly alter the physical structure of the bank. Farming on the river terrace is likely to result in agricultural pollution of river water and bank erosion. Scores were higher for places where there was less waste and farmland. The width-depth ratio may reflect channel stability; channels with a lower width-depth ratio were more stable [27], and received a high score.

The detailed scoring scheme is shown in the Appendix. A five-point scoring method was applied to give each indicator a primary score. Each indicator was classified into five grades that were scored between 1 and 5 points. The total score of the indicators represented the score for a site. Primary score were then translated into final score of a site using following equation: Q = $\left(\overset{\text{n}}{\underset{\text{i}=1}{\sum }}\text{Si}\right)/\left(5\times \text{n}\right)$, where Q was the total score, Si represented the score of the ith indicator, and n was the number of indicators, which in this case was 10. Thus, the final score for a site was between 0 and 1. By applying K-means cluster analysis, the final scores for all the sites were divided into five categories to reflect the variation in habitat quality. RHQ was categorized into five grades, including excellent, good, moderate, poor, and bad.

#### 2.2.3. Field Survey

An accurate field survey can ensure an authentic evaluation. Instruments were used to measure quantitative indicators (e.g., a laser rangefinder was used to measure length, height, and angle). To account for the heterogeneity of indicators along rivers, three replicate measurements were made at each site. The length of a surveyed reach was five times the mode bank-full width, and minimum and maximum lengths were 100 m and 500 m, respectively. At each site, basic information about the sampling sites relating to the basin and reach was recorded first. This information included, among other variables, the location code, longitude and latitude, altitude, administrative district, date and time, and weather. After this was completed, the following detailed investigations were carried out on both the left and right banks of the channel.

##### Width of Riparian Zone

The vertical distance from the edge of the stream to the point where artificial land use (except artificial vegetation for environmental improvement) started was measured by a laser rangefinder [28].

##### Land Use Pattern

Land use patterns beyond the immediate riparian zone were recorded.

##### Coverage of Riparian Vegetation

In the mountain areas of the HRB, some of the riparian zones were partially covered with natural and near-natural trees, shrubs, and grasses. In the plain areas, however, there was no natural vegetation, and herbs comprised annual or biannual common field weeds. Given that trees and shrubs are good vegetation types and play an important role in bank protection [29], tree and shrub (excluding grass) coverage was measured in the plain areas. In the riparian zones, the tree and shrub canopy cover was measured using a laser rangefinder and tape. Overhead photos of 1 m^2^ areas of grassland were taken with a digital camera and were processed with Adobe Photoshop 6.0 software to obtain the canopycover. At least ten photos were taken at each site. The grass coverage was measured in areas that were outside of the tree or shrub canopy cover. The percentage of the total canopy cover of trees, shrubs, and grasses served as the figure for the final coverage.

##### Continuity of Riparian Vegetation

In mountain areas, vegetation was classified as semi-continuous if the vegetation on the left and right sides of a surveyed reach was very obviously disconnected by an access path to the stream. In some areas there was more intensive human activity, such as mining, which left large areas of bare land and only small patches of vegetation. In the worst cases, the distribution pattern of vegetation was scattered and isolated. In other cases, the vegetation distribution was continuous. In plain areas, vegetation surveys focused on artificial trees and shrubs that were often arranged in rows. If an interval appeared along the channel that was less than three times the average spacing of the other trees, the vegetation was defined as semi-continuous. If the percentage of the length of bare land exceeded 50%, it was described as discontinuous. Alternatively it may have been scattered and isolated, which was easy to identify, or it may have been continuous.

##### Bank Modification

This indicator represented farming and solid waste on the bank slope. The former was qualitative and observable. The volume of solid waste was measured using a tape measure on non-rainy days.

##### Channel Structure

A laser rangefinder and measuring tapes were used to measure the width:depth ratio. Width referred to the bank-full width of the channel, while the depth was the sum of the depth of the water and the height of the bank.

##### Channel Substrate

River substratum (to a depth of 10 cm) included the following substrate categories: bedrock, boulders (>256 mm), cobbles (64–256 mm), pebbles (16–64 mm), gravel (2–16 mm), sand (0.06–2 mm), silt (0.004–0.06 mm), and clay (<0.004 mm). Line transects were used to measure the occurrence frequency of each substrate type. A steel tape was placed randomly across substratum, and then photos were taken vertically by a digital camera. At least 20 sampling lines (each line was 2 m long) were obtained in each surveyed reach. Substrate types that had an occurrence frequency above 50% were recorded at a site.

##### Channel Pattern

This was directly observed, and included engineered single flow channels, single flow channels, distributary channels, and braided channels.

##### Hydrophyte Richness

This was assessed by moving in the direction of flow, and observing the aquatic plants in transects at 5-m intervals. At least 20 transects were taken within each surveyed reach. Plants that had an occurrence frequency above 50% were recorded at a site. The plants recorded included emergent plants, submerged plants, leaf floating plants, floating plants, algae, and moss.

##### Coverage of Emergent Plants

A laser rangefinder and tape were used to assess the coverage of emergent plants along the channel.

#### 2.2.4. Data Analysis

Data included field surveys of river habitats and socio-economic data of 25 prefecture-level cities and two municipalities of the HRB that were obtained from the Statistical Yearbooks of 1985–2009. A digital raster map of the HRB was provided by the Institute of Remote Sensing Applications, Chinese Academy of Sciences. The locations of reservoirs and sluices were determined from the Chinese Code for Water Gate Names and The Hai River Basin, Volume 1 [30,31]. Remote sensing images from 2005 were interpreted to obtain land use types. Water resource data were collected from the Water Resources Protection Bureau of the HRB and the Water Resources Bulletin of the HRB. Basic analysis of raw data (e.g., standard deviations, summary statistics) was done with Microsoft Excel 2007. More advanced analysis, such as K-means cluster analysis for classifying the RHQ scores, was performed using SPSS 17.0. ArcGIS 10.0 was used to present the results of the RHQ assessment, and the distribution of the population density, cities, land use types, and industrial production.

In our assessing method, k-means clustering was used to divide scores into bands representing different levels of ecological condition. k-means clustering aims to partition n observations into k partitions (clusters) in which each observation belongs to the cluster with the nearest mean, serving as a prototype of the cluster. k-means clustering, which is efficient and succinct, has been most widely used. The longitudinal connectivity of river system in the HRB can be estimated by the index of connectivity [32]. The equation for this index is C = S/N, in which S represents the length of the river network and N represents the quantity of hydraulic structures.

## 3. Results and Discussion

### 3.1. River Habitat Quality

**Figure 2:**
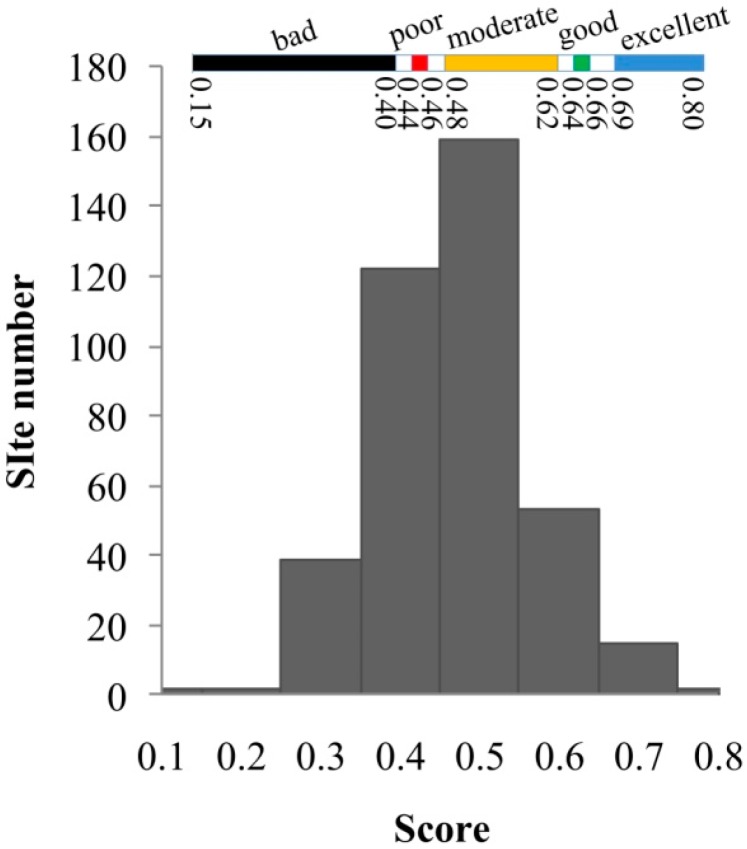
Distribution of assessed score values.

**Figure 3:**
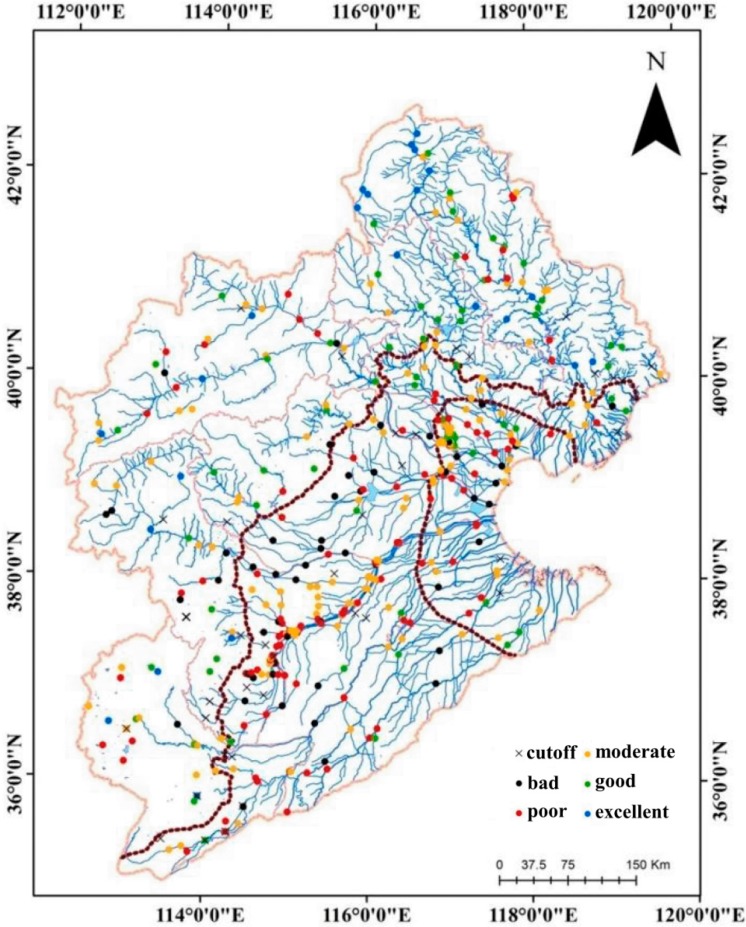
Distribution of RHQ in HRB.

The river habitat status in the HRB is alarming. The assessment scores were normally distributed and ranged from 0.15 to 0.80 (Figure 2). The value interval with the highest frequency was 0.4–0.6, and included 281 sites. The bad class had the largest score interval (0.15–0.40) (Figure 2). Most surveyed sites were in the moderate RHQ category, which ranged from 0.48–0.62. The ranges of scores for good and poor were minimal. The score distributions indicated that the RHQ of the HRB was generally from “moderate” to “poor”. The highest proportion (34.56%) of sites was classified as “moderate”, whereas 26.72% and 15.69% of sites were classified as “poor” and “bad”, respectively (Figure 3, Table 1). However, 5.64% and 17.4% were classified as “excellent” and “good”, respectively, which indicates that over 40% of the rivers in the HRB need rehabilitation and restoration, and only 20% of the RHQ is in good condition. In addition, the moderate group represents a vulnerable habitat condition, and shows that over 30% of the river habitat will probably degrade further under human disturbance. RHQ in the upper mountain zone was better than that in the middle plain and littoral areas (Figure 3; Table 1 and Table 2). Up to 15.86% of the upper zone sampling sites were classified as “excellent”, and 6.21% of the sites were “bad”. In contrast, the middle plain and littoral areas had no “excellent” sampling sites, but contained about half of the “poor” to “bad” sampling sites.

**Table 1:** Proportion of sites of different river habitat quality in the river systems.

River system	Excellent	Good	Moderate	Poor	Bad
Luan	21.43%	30.36%	28.57%	17.86%	1.79%
Ziya	2.79%	6.51%	37.21%	28.37%	25.12%
Tuhaimajia	0.00%	23.81%	23.81%	38.10%	14.29%
Daqing	0.00%	16.22%	29.73%	21.62%	32.43%
Beisan	2.56%	23.07%	41.02%	21.79%	11.54%
Yongding	9.37%	18.75%	28.13%	28.13%	15.63%
Zhangwei	8.11%	16.22%	43.24%	24.32%	9.11%
Heilonggang	0.00%	12.90%	25.81%	45.16%	16.13%
Total basin	5.64%	17.40%	34.56%	26.72%	15.69%

**Table 2:** Proportion of sampling sites in the different RHQ classes in the three zones.

Zones	Excellent	Good	Moderate	Poor	Bad
Upper mountain area	15.86%	28.97%	32.41%	16.55%	6.21%
Middle plain area	0.00%	8.70%	37.89%	32.92%	20.49%
Littoral area	0.00%	15.71%	38.57%	30.00%	15.71%

### 3.2. Differential Representation of the RHQ in the Three Zones

The profile of river habitat condition in the HRB is presented in Table 3. The scores were very different for the three main physiographic sections. Generally, the upper mountain area had more advantages, such as wider riparian zones, high coverage of riparian vegetation, rare farming on banks, and low volumes of solid waste. The middle plain and the littoral areas demonstrated many characteristics of poor condition, such as narrow riparian zones, artificial land use types, low coverage of riparian vegetation, large amounts of solid waste, and farming on the bank slopes. River habitat degradation was defined by different characteristics in the three zones. Sites with poor and bad habitat conditions were selected and the main factors that contributed to habitat degradation were analysed (Table 3 (in italics)). The single channel pattern and lower hydrophyte richness resulted in river habitat degradation in the upper mountain area. Primary scores ≤2 were reported for channel pattern and hydrophyte richness at 77% and 87% of the poor and bad sites, respectively. There were similarities between the characteristics that caused habitat degradation and negatively influenced the RHQ in the middle plain area and the littoral area. These included the width of the riparian zone, riparian vegetation coverage, artificial land-use pattern, frequent occurrence of farming and solid waste on the river banks, single channel flow pattern, and rare aquatic plants. Intensive human activities along rivers in the plain areas (including the middle plain area and the littoral area) have very obviously destroyed the riparian habitat structure; rivers with visibly black water caused by excessive pollutant discharges serve as a hell for aquatic plants.

**Figure 4:**
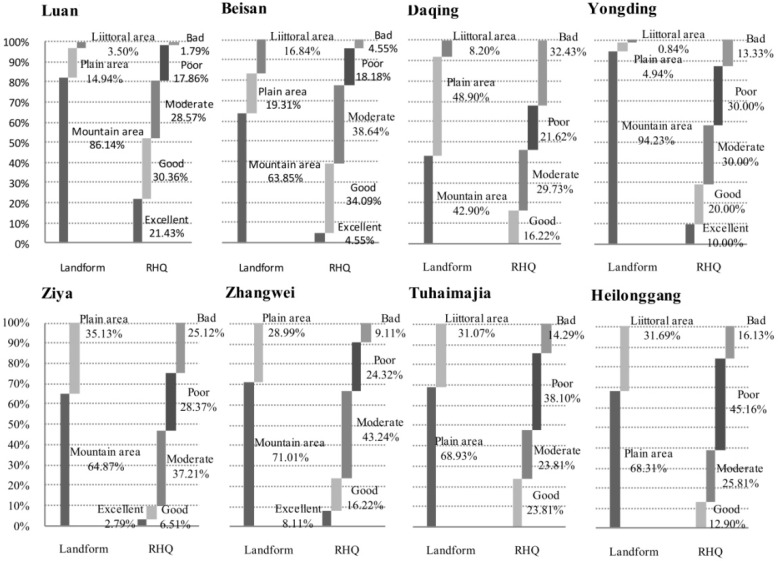
Percentage of different land forms and RHQ in each secondary river system.

The RHQ of each secondary river system was related to the relative percentage of mountain and plain area (Figure 4). The Luan, Yongding, and Beisan Rivers, with more than 30% of the excellent and good sampling sites, had better habitat quality than the remaining river systems. RHQ was the lowest in the Ziya and Heilonggang Rivers, which together had less than 15% of the excellent and good sampling sites (Table 1).

**Table 3:** Indicators of river habitat condition in the HRB.

	Upper Mountain Area	Middle Plain Area	Littoral Area
Width of riparian zone	14.50–30.00 m	0.00–30.00 m. *75% of “poor”&“bad” sites with primary score* ≤ *2*	2.50–30.00m. *66% of “poor”&“bad” sites with primary score* ≤ *2*
Land-use pattern	Mainly near-natural vegetation	Mainly farmland and public land. *63% of “poor”&“bad” sites with primary score* ≤ *2*	Mainly public land and industrial land. *69% of “poor”&“bad” sites with primary score* ≤ *2*
Coverage of riparian vegetation	23%–81%	2%–55%. *82% of “poor”&“bad” sites with primary score* ≤ *2*	1%–30%. *86% of “poor”&“bad” sites with primary score* ≤ *2*
Continuity of riparian vegetation	Continuous/semi-continuous patches	Continuous/semi-continuous rows and small patches, discontinuous small patch, scattered and isolated	Semi-continuous rows and small patches, scattered and isolated
Bank modification	Rarely farming on bank; volume of solid waste was 1–5 m^3^	Commonly farming on bank; volume of solid waste was 1–120 m^3^. *58% of “poor”&“bad” sites with primary score* ≤ *2*	Rare farming on bank; volume of solid waste was 1–110 m^3^. *56% of “poor”&“bad” sites with primary score* ≤ *2*
Width/depth ratio	2.58–217.00	3.12–150.00	6.18–56.67
Channel substrate	Mainly boulder, cobble, pebble gravel; rarely bedrock, sand, silt and clay	Mainly silt and clay and hewn stones; rarely sand, and gravel	Mainly sand, silt and clay. *87% of “poor”&“bad” sites with primary score* ≤ *2*
Channel pattern	Distributary, braided and single flow channel. *77% of “poor”&“bad” sites with primary score* ≤ *2*	Mainly single flow channel, rarely distributary and hardened channel. *67% of “poor”&“bad” sites with primary score* ≤ *2*	Mainly single flow channel, rarely distributary channel. *81% of “poor”&“bad” sites with primary score* ≤ *2*
Hydrophyte richness	Emergent plant, submerged plant, algae and moss. *87% of “poor”&“bad” sites with primary score* ≤ *2*	Mainly emergent plant, submerged plant, floating plant; rarely algae and moss. *62% of “poor”&“bad” sites with primary score* ≤ *2*	Mainly emergent plant, submerged plant. *83% of “poor”&“bad” sites with primary score* ≤ *2*
Coverage of emergent plants	3%–72%	1%–90%. *69% of “poor”&“bad” sites with primary score* ≤ *2*	0%–78%. *78% of “poor”&“bad” sites with primary score* ≤ *2*

The river systems with larger areas of mountain had a higher percentage of excellent and good RHQ scores, such as the Luan, Beisan, and Yongding. The Tuhaimajia and Heilonggang Rivers, with no mountain areas, had a lower percentage of sites with high river quality. There were sites with poor and bad RHQ scores in each river system, reflecting widespread degradation of the river habitat throughout the entire HRB. The percentage of sites with poor and bad RHQ was lower in the Luan and Beisan Rivers than in other river systems, and this reflects the fact that the mountain areas of the Luan River cover northern Hebei province and part of the Inner Mongolian Plateau, which have less economic development, lower population densities, and less intensive land use than the other river systems. The river habitats in the mountain areas around Beijing received better protection.

River habitat quality and its special distribution were revealed clearly, however, further justification needed. Unfortunately, there is a lack of research on river habitat quality or degradation in the HRB and its secondary basins. Using a Fuzzy Matter-element Model, Liu *et al.* [33] assessed the ecological status of the Luan river, but not the whole basin. Indicators included river form, flow, water quality, riparian attributes, phytoplankton, habitat (soil and wetlands loss), water supply, and flood safety. Though their research had a differential angel and a much grander scale, results showed a relatively good environment in the Luan river, which indicated a potential consistency with our research result and robustness of our assessing method.

### 3.3. Discussion on Controlling Factors of RHQ

**Figure 5:**
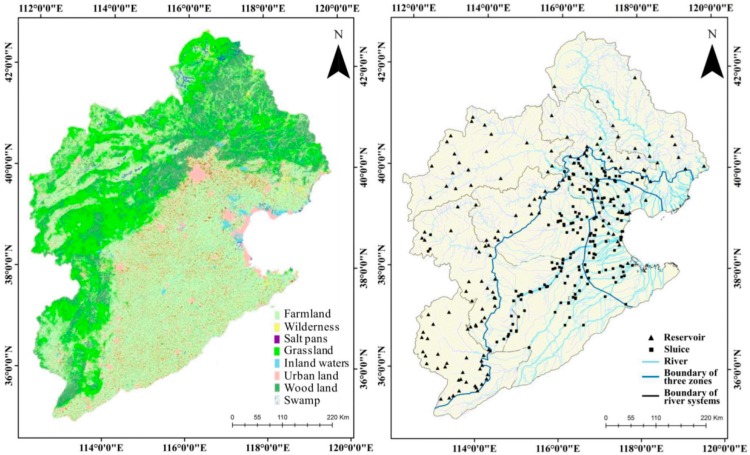
Distribution of land use types and reservoirs and sluices in HRB.

Further analysis showed that human pressures on river ecosystems had a significant impact on RHQ, based on the principle that population density, city distribution, industrial production, and land use type were significantly different between mountain areas and plain areas (Figure 5 (left), Table 4). Compared with the mountain areas, the plain areas had higher population densities, more intensive urban agglomerations, and higher industrial production value. Land use in the mountain areas was mainly woodland and grassland. However, while the plain areas were dominated by farmland, there were other land use types such as swamps, inland waters, salt pans, construction land, and urban land. We analysed the relationships between RHQ and GDP, population, and several major land use types, such as woodland, grassland, agricultural land, construction land, and urban land (Table 4). The results showed that RHQ was significantly and positively correlated with woodland and grassland, and was significantly and negatively correlated with agricultural land and urban land. There were no significant correlations between RHQ and GDP, population, and construction land, which showed that these three factors had no direct influence on RHQ. In addition, moderate RHQ was not significantly correlated with these factors, which indicates the accuracy of assessment.

**Table 4:** Correlation analysis of RHQ scores with several factors.

	Woodland	Grassland	Farm Land	Construction Land	Urban Land	GDP	Population
Excellent	1.000 **	1.000 **	−0.985	−0.807	−0.962	−0.474	−0.413
good	−0.936	−0.941	−0.985	−0.558	−0.997 *	−0.746	−0.698
moderate	−0.996	−0.995	0.963	0.862	0.930	0.383	0.319
poor	−0.984	−0.986	1.000 **	0.697	0.994	0.615	0.559
bad	−0.940	−0.945	0.987	0.568	0.998 *	0.738	0.689

Note: * indicates a significant correlation at 0.05 level (*p* < 0.05); ** indicates a significant correlation at 0.01 level (*p* < 0.01).

Assemblages of urban land and farmland greatly disrupt the lateral connectivity of the river system. In the plain area, agricultural land represented up to 83% of the land (Figure 5 (left)). The riparian area is occupied by vast areas of agricultural land, impeding the communication between aquatic and terrestrial organisms and reducing the river flood storage function. This agrees with the findings from our field survey, where we observed that farmland was often adjacent to river channels, especially in the plain area. The riparian zone plays an important role in the regulation of aquatic-terrestrial linkages and their manipulation is a common watershed management tool in disturbed ecosystems [34,35]. Loss of riparian zone has had serious negative impacts on the river habitat of the HRB.

Connectivity along the channel has been blocked by a large number of reservoirs built throughout history (Figure 5 (right)). With the establishment of the New China, construction of water conservation facilities developed rapidly. By 1985, 1967 reservoirs, including large-, medium-, and small-sized reservoirs, had been built in the HRB, with a storage capacity of 26.4 billion·m^3^. As much as 81% of the rivers in the mountain area were controlled [30]. Large amounts of water storage in reservoir strips at the boundary of the mountains and plains directly cut off the connectivity between the mountain and plain areas and have contributed to water shortages in the plain areas. A total of 427 sluices were built, mainly in the plain area (Chinese Code for Water Gate Names, 2000) (Figure 5 (right), Table 5), which immediately decreased the flow velocity. Combined with the inherent water shortage of the HRB, cut-off and dried-up rivers have become a common phenomenon in the plain area. The longitudinal connectivity in the HRB can be estimated by the index of connectivity [32]. Index values for the river systems in the HRB ranged from 2.08 to 16.56 (Table 5). The Yongding River system had the minimum index value, followed by the Daing, Beisan, Ziya, Luan, and Zhangwei Rivers. The index value exceeded 10 in only two river systems. Poor channel connectivity disrupts natural hydrologic cycle and has a negative impact on the RHQ. Natural continuous flow is replaced by pulse discharge which easily modifies bank form below a dam or sluice. In plain area, stagnant water appears frequently due to intercept of flow by sluices, which contributes to high levels of pollution, followed by large amount of polluted sediment in channel and poor aquatic plant community.

**Table 5:** Index of river continuity in the Hai River Basin.

River System	Quantity of Dams and Sluices	Length of River Network (km)	Index of Connectivity
Luan	449	1653.50	3.68
Beisan	257	967.20	3.76
Yongding	589	1223.60	2.08
Daqing	172	430.50	2.50
Ziya	438	1622.80	3.70

To sum up, intensive artificial land use induced by urbanization is the main factor that has impacted on the RHQ of the HRB. Farmland and urban land occupy the riparian area, and have blocked the rivers’ lateral connectivity. In addition, a large number of reservoirs and sluices have disrupted the rivers’ longitudinal connectivity, which has exacerbated river habitat degradation.

## 4. Conclusions

We used a scoring system to assess the RHQ in the HRB. From this analysis, we arrived at the following important conclusions: (1) The RHQ of the entire HRB is poor. Specifically, 42.41% of the sites, accounting for 3.31 × 10^4^ km of river length, had poor and bad habitats. Habitats are most seriously deteriorated in the plain areas, and nearly 50% of the sites, accounting for a river length of 1.65 × 10^4^ km, had poor and bad habitats. Narrower riparian zones (≤5 m), lower coverage of riparian vegetation (≤40%), artificial land use patterns (public and industrial land), frequent farming and solid waste (nearly 10 m^3^) on the river banks, single channel flow pattern, and rare aquatic plants (≤1 category) were the main contributors to river habitat degradation. (2) At the regional scale, intensive artificial land use induced by urbanization was the main factor that impacted on the RHQ in the HRB. Farmland and urban land occupy the riparian area, disrupting the lateral connectivity of the rivers; the large number of reservoirs and sluices cut off the longitudinal connectivity of the rivers. Priority should be given to reconstructing the river habitat in the HRB, especially in the plain areas, and should be based on adjusting the land use structure and the regulation of reservoirs and sluices. In addition, there should be enhanced protection of the river habitat in the upstream catchment areas.

## Appendix

**Table A1:** Scoring system for assessment of river habitat in the Hai River Basin.

Site Number				River Name				Location					Catchment	
Date & Time				Surveyor Name				Weather Conditions					Flow Conditions	
										Score	Notes			
1. Width of riparian zone from stream edge to field														
Marshy or woody riparian zone >30 m wide										5 □				
Marshy or woody riparian zone varying from 15 to 30 m										4 □				
Marshy or woody riparian zone varying from 5 to 15 m										3 □				
Marshy or woody riparian zone 1 to 5 m										2 □				
Marshy or woody riparian zone absent										1 □				
2. Land-use pattern beyond the immediate riparian zone														
Near-natural, consisting of grassland, forest, bushes										5 □				
Artificial vegetation, consisting of woodland, bushes										4 □				
Farmland, cropland, orchard										3 □				
Public Land, consisting of parks, roads, houses, buildings										2 □				
Industrial plants, livestock farms, mines										1 □				
3. Coverage of riparian vegetation											To investigate trees, shrubs, and natural grassland, excluding annual and biennial field weeds. To record species of trees and/or shrubs			
80%–100%										5 □				
60%–80%										4 □				
40%–60%										3 □				
20%–40%										2 □				
0%–20%										1 □				
4. Continuity of riparian vegetation											To investigate trees, shrubs, and natural grassland, excluding annual and biennial field weeds. To record species of and/or shrubs			
Continuously large patches										5 □				
Continuously rows and/or small patches										4 □				
Semi-continuous rows										3 □				
Discontinuously small patches										2 □				
Scattered and isolated										1 □				
5. Bank modification											Subtract 1 point if reinforced bank exists			
Solid waste < 1m^3^ and no farming on bank slope										5 □				
1 m^3^ ≤ solid waste < 3 m^3^ and no farming on bank slope										4 □				
3 m^3^ ≤ solid waste < 10 m^3^ and no farming on bank slope										3 □				
Solid waste < 10 m^3^ and farming on bank slope										2 □				
Solid waste ≥ 10 m^3^ and farming on bank slope										1 □				
6. Channel structure														
Width/depth < 12										5 □				
Width/depth 12–40										4 □				
Width/depth 40–100										3 □				
Width/depth 100–200										2 □				
Width/depth > 200										1 □				
7. Channel substrate											Substrate classes including (1) bedrock, boulder and/or cobble; (2) pebble and/or gravel; (3) sand; (4) silt and clay. To add 1 point for each rare feature (woody debris, hewn stones only for plain rivers).			
Four classes of substrate appear simultaneously										5 □				
Three classes appear										4 □				
Two classes appear										3 □				
Only one kind of substrate appears										2 □				
Hardened cement, being different from the above classes										1 □				
8. Channel pattern														
-										5 □	Add 1 point if fallen trees or riffle and/or pool appear			
Braided channel										4 □				
Distributary channel										3 □				
Single flow channel										2 □				
Hardened single flow channel										1 □				
9. Hydrophyte richness											Hydrophytes contain emergent plant, submerged plant, leave floating plant, floating plant, and algae, moss.			
Four categories										5 □				
Three categories										4 □				
Two categories										3 □				
One category										2 □				
none										1 □				
10. Coverage of emergent plants along channel														
>70%										5 □				
50%–70%										4 □				
30%–50%										3 □				
10%–30%										2 □				
<10%										1 □				
